# Clinical phenotyping in sarcoidosis using cluster analysis

**DOI:** 10.1186/s12931-022-01993-z

**Published:** 2022-04-09

**Authors:** Nancy W. Lin, Jaron Arbet, Margaret M. Mroz, Shu-Yi Liao, Clara I. Restrepo, Annyce S. Mayer, Li Li, Briana Q. Barkes, Sarah Schrock, Nabeel Hamzeh, Tasha E. Fingerlin, Nichole E. Carlson, Lisa A. Maier

**Affiliations:** 1 Division of Pulmonary Sciences and Critical Care Medicine, Department of Medicine, University of Colorado, Aurora, CO USA; 2 Division of Environmental and Occupational Health Sciences, Department of Medicine, National Jewish Health, Denver, CO USA; 3 Colorado School of Public Health, Aurora, CO USA; 4 Department of Medicine, University of Iowa, Iowa City, IA USA; 5 Center for Genes, Environment & Health, National Jewish Health, Denver, CO USA; 6 Department of Immunology and Genomic Medicine, National Jewish Health, Denver, CO USA; 7 Division of Environmental and Occupational Health Sciences, National Jewish Health G211, 1400 Jackson Street, Denver, CO 80206 USA

**Keywords:** Cluster analysis, Disease severity, Phenotypes, Pulmonary, Sarcoidosis

## Abstract

**Background:**

Most phenotyping paradigms in sarcoidosis are based on expert opinion; however, no paradigm has been widely adopted because of the subjectivity in classification. We hypothesized that cluster analysis could be performed on common clinical variables to define more objective sarcoidosis phenotypes.

**Methods:**

We performed a retrospective cohort study of 554 sarcoidosis cases to identify distinct phenotypes of sarcoidosis based on 29 clinical features. Model-based clustering was performed using the VarSelLCM R package and the Integrated Completed Likelihood (ICL) criteria were used to estimate number of clusters. To identify features associated with cluster membership, features were ranked based on variable importance scores from the VarSelLCM model, and additional univariate tests (Fisher’s exact test and one-way ANOVA) were performed using q-values correcting for multiple testing. The Wasfi severity score was also compared between clusters.

**Results:**

Cluster analysis resulted in 6 sarcoidosis phenotypes. Salient characteristics for each cluster are as follows: Phenotype (1) supranormal lung function and majority Scadding stage 2/3; phenotype (2) supranormal lung function and majority Scadding stage 0/1; phenotype (3) normal lung function and split Scadding stages between 0/1 and 2/3; phenotype (4) obstructive lung function and majority Scadding stage 2/3; phenotype (5) restrictive lung function and majority Scadding stage 2/3; phenotype (6) mixed obstructive and restrictive lung function and mostly Scadding stage 4. Although there were differences in the percentages, all Scadding stages were encompassed by all of the phenotypes, except for phenotype 1, in which none were Scadding stage 4. Clusters 4, 5, 6 were significantly more likely to have ever been on immunosuppressive treatment and had higher Wasfi disease severity scores.

**Conclusions:**

Cluster analysis produced 6 sarcoidosis phenotypes that demonstrated less severe and severe phenotypes. Phenotypes 1, 2, 3 have less lung function abnormalities, a lower percentage on immunosuppressive treatment and lower Wasfi severity scores. Phenotypes 4, 5, 6 were characterized by lung function abnormalities, more parenchymal abnormalities, an increased percentage on immunosuppressive treatment and higher Wasfi severity scores. These data support using cluster analysis as an objective and clinically useful way to phenotype sarcoidosis subjects and to empower clinicians to identify those with more severe disease versus those who have less severe disease, independent of Scadding stage.

**Supplementary Information:**

The online version contains supplementary material available at 10.1186/s12931-022-01993-z.

## Background

Sarcoidosis is a heterogeneous disease, affecting any organ and with variable natural history [1, 2]. Clinical phenotyping in complex diseases such as sarcoidosis can help define subpopulations with similar clinical/biological characteristics. Most importantly, phenotyping may differentiate disease course, identifying those with worse prognosis requiring long-term treatment and follow-up [3]. Based on previous studies, several characteristics portend worse prognosis in sarcoidosis, including race, Scadding stage, BMI, treatment status and lung function [4–7]. However, transforming these characteristics into discrete and validated sarcoidosis phenotypes, especially ones with clinical implications for disease status and prognostication, has proved challenging.

Several phenotyping classifications have been proposed in sarcoidosis. Most rely on expert opinion even though this way may introduce bias, which can limit agreement between experts and consistency of application. An example of expert opinion based phenotyping was proposed in Wasfi et al., where a disease severity score was derived from subjective assessments by sarcoidosis experts [8]. A benefit of the Wasfi score is the ease of obtaining inputs at one clinic visit to determine phenotype/severity. A limitation is that it has not been externally validated; however, the severity score was internally validated by an independent panel of international experts within the study. Additionally, several studies have used the Wasfi score as a way to measure sarcoidosis severity [9, 10]. Recently, cluster analysis has been used to determine phenotypes in many complex diseases. Cluster analysis employs multivariate algorithms to organize individuals into subgroups based on similarities [11, 12]. The clustering methodology is considered relatively unbiased since it employs objective statistical methods to group individuals rather than expert opinion; however, selection of input variables is still a subjective process. Schupp et al., Rubio-Rivas et al. and Lhote et al. have used cluster analysis to subgroup organ involvement in sarcoidosis [13–15]. However, these phenotypes do not necessarily provide information on disease severity or prognosis and can be difficult to apply in a single clinic visit versus multiple visits over time.

We propose that cluster analysis can be used to identify clinical phenotypes of sarcoidosis including severe and less severe forms of the disease. In this study we use this technique and include clinical variables that have influenced prognosis in previous studies. We will also associate resultant phenotypes with the Wasfi severity score to assess differences in disease severity between clusters. Some of the results of this study have been previously reported in the form of an abstract [16].

## Methods

### Study population

This was a cross-sectional, retrospective study on sarcoidosis cases seen in the Division of Occupational and Environmental Health Sciences at National Jewish Health (NJH) from 2008 to 2015, enrolled as part of a substudy to an NIH funded genetic study (R01HL11487, manuscript in preparation). All subjects provided written informed consent to participate in this study. The study was approved by the NJH Institutional Review Board (HS 2458).

All sarcoidosis subjects met the American Thoracic Society/European Respiratory Society criteria for the diagnosis of sarcoidosis including tissue biopsy confirmation [2]. Medical charts were reviewed to ensure eligibility and extract clinical data. All subject information was collected at the reference enrollment date, defined as the time of spirometry and chest x-ray, except for treatment as noted below. If only spirometry was available, then that date was used for enrollment.

Gender, race, BMI and smoking status were collected at enrollment. The FVC% predicted (FVCpp), FEV1% predicted (FEV1pp), and FEV1/FVC ratio (%) were included in the analysis. For interpretation of spirometry data, we considered normal to be ≥ 80% FEV1pp and FVCpp and ≥ 70% FEV1/FVC as we did not have lower-limit-of-normal available for all participants [17]. Scadding stages were determined by the interpreting radiologist from chest x-rays closest to enrollment. Biopsy dates were recorded if available and used to determine duration of disease and age at diagnosis.

Organ involvement was determined based on the WASOG Sarcoidosis Organ Assessment Instrument [18]. Our sarcoidologists NH, LAM, SYL, CIR reviewed all cases and assigned sarcoidosis organ involvement for organs that met the “highly probable” and “at least probable” classification outlined in the WASOG instrument. Those presenting with traditional signs of Lofgren’s syndrome were noted.

Treatment was defined as being on non-corticosteroid immunosuppressive therapy including methotrexate, azathioprine, mycophenolate mofetil, leflunomide, infliximab, and adalimumab. Hydroxychloroquine was not considered systemic treatment given its nonspecific indications. Treatment with corticosteroids, i.e., prednisone, was not included since some individuals are placed on steroids at diagnosis without a clinical indication. A dichotomous variable indicating the presence or absence of therapy up to 5 years after the enrollment date was included; this time frame was chosen to approximate those who were ever versus never treated.

### Wasfi severity score

The sarcoidosis severity score, adapted from Wasfi et al. [8], was calculated for each individual using the following equation:

$${\text{Severity score}} = {11}.{46} + {3}.{9}\left( {\text{C}} \right) + {2}.{56}\left( {\text{N}} \right) + {1}.{56}\left( {{\text{IS}}} \right) - 0.0{51}\left( {{\text{FVC}}\% {\text{ predicted}}} \right) + {1}.{75}\left( {{\text{AA}}} \right) - 0.0{54}({\text{FEV1}}/{\text{FVC}})$$

C = 1 for cardiac; N = 1 for neurological; IS = 1 if individual received non-corticosteroid immunosuppression within 30 days of enrollment date; AA = 1 for African American. Missing data was coded as a 0.

### Statistical analysis

All statistical analyses were performed using R (R Core Team, 2020) [19]. Model-based clustering was used to identify sarcoidosis phenotypes based on features shown in Table 1. Variations of the model included a single dichotomous extrapulmonary variable (absence or presence of extrapulmonary disease) versus individual organs. Clustering was performed using the VarSelLCM R package [20]. We chose VarSelLCM given that it supports mixed types of features, missing values, and variable selection to identify important clustering features [21]. VarSelLCM handles missing values using an expectation maximization algorithm. Simulations in Marbac et al. show that the methods work well even when variables have up to 20% missing values [20]. The Integrated Completed Likelihood (ICL) criterion was used to estimate the number of clusters [22]. To identify features associated with cluster membership, variables were ranked based on the variable importance scores from the VarSelLCM model, and additional univariate tests (Fisher’s exact test (FET) and one-way ANOVA) were performed. Pairwise comparisons were made between clusters using 2-sample t-tests for quantitative features and logistic regression for categorical features (FET as appropriate). To account for multiple testing, the Benjamini–Hochberg method was used to calculate false-discovery-rate (FDR) adjusted p-values, hereby referred to as ‘q-values.’ [23] Results with q-values < 0.05 were considered statistically significant.

**Table 1:** Characteristics of the Study Population

Characteristic	Missing: N (%)	N = 554^1^
Gender		
Male	0 (0.0)	265 (47.8%)
Female		289 (52.2%)
Race		
White	12 (2.2)	443 (81.7%)
Black		90 (16.6%)
Asian		4 (0.7%)
American Indian		2 (0.4%)
Other		3 (0.6%)
Smoking status		
Never smoker	2 (0.4)	367 (66.5%)
Ever smoker		185 (33.5%)
Lungs	0 (0.0)	534 (96.4%)
Cardiac	0 (0.0)	71 (12.8%)
Skin	0 (0.0)	68 (12.3%)
Eye	0 (0.0)	58 (10.5%)
Calcium/Vit D Metabolism	0 (0.0)	54 (9.7%)
Liver	0 (0.0)	40 (7.2%)
Extrathoracic lymph nodes	0 (0.0)	35 (6.3%)
Bone-Joint	0 (0.0)	19 (3.4%)
Spleen	0 (0.0)	19 (3.4%)
Lofgren’s	0 (0.0)	17 (3.1%)
Other organs	0 (0.0)	17 (3.1%)
Neuro	0 (0.0)	14 (2.5%)
ENT	0 (0.0)	12 (2.2%)
Kidney	0 (0.0)	9 (1.6%)
Muscle	0 (0.0)	7 (1.3%)
Parotid salivary	0 (0.0)	5 (0.9%)
Bone marrow	0 (0.0)	5 (0.9%)
Extrapulmonary involvement	0 (0.0)	259 (46.8%)
Ever treatment	104 (18.8)	310 (68.9%)
Scadding stage		
0	44 (7.9)	102 (20.0%)
1		67 (13.1%)
2		183 (35.9%)
3		86 (16.9%)
4		72 (14.1%)
Number of organs involved		
0	0 (0.0)	10 (1.8%)
1		303 (54.7%)
2		149 (26.9%)
3		53 (9.6%)
4		24 (4.3%)
5		14 (2.5%)
6		1 (0.2%)
Mean number of organs	0 (0.0)	1.7 (1.0)
BMI	0 (0.0)	30.5 (6.8)
Age at diagnosis	1 (0.2)	46.6 (10.9)
Duration of Disease	1 (0.2)	6.0 (7.1)
FEV1/FVC	0 (0.0)	74.9 (9.8)
FVCpp	0 (0.0)	86.7 (16.8)
FEV1pp	0 (0.0)	83.2 (19.4)

^1^Data presented: n (%); mean (SD)

## Results

### Characteristics of study population

The characteristics of our study population, consisting of 554 individuals (Table 1), reflect a slight female majority and more White individuals, although there was a greater percentage of Black individuals than would be expected based on the racial breakdown of Colorado. The lungs were most commonly involved (96.4%) with Scadding stage 2 most prevalent. Next most frequently involved organs included cardiac (12.8%), skin (12.3%) and eye (10.5%). Most individuals had only one organ involved (54.7%). Most cases (68.9%) were treated with non-corticosteroid immunosuppression within 5 years of enrollment.

### Cluster analysis defines six phenotypes

**Fig. 1:**
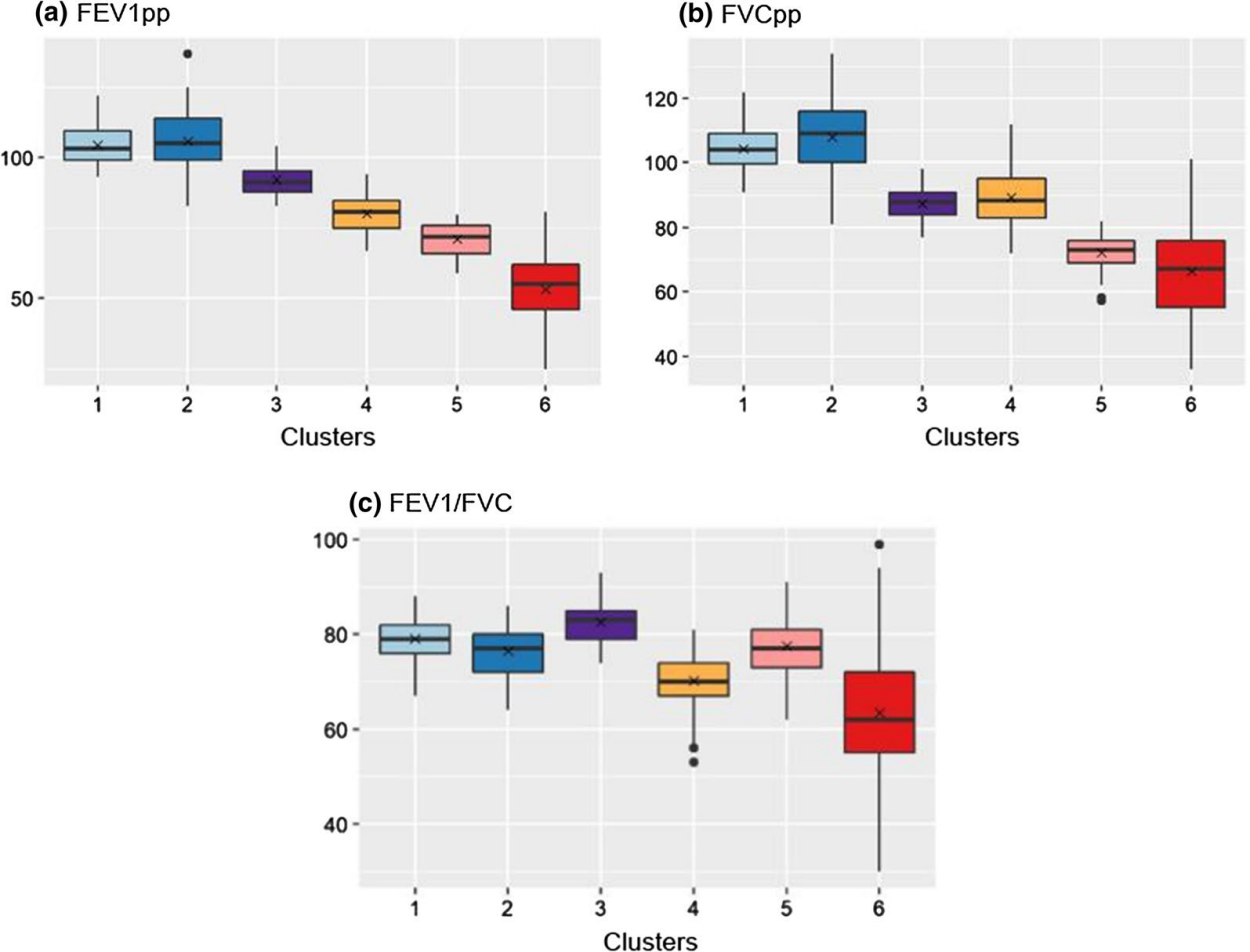
Comparison of lung function parameters among clusters. For each cluster, median and IQR are shown by boxplots and means are shown by x in the center of boxplots for **a** FEV1pp **b** FVCpp and **c** FEV1/FVC. Potential outliers are indicated by distinct points

**Fig. 2:**
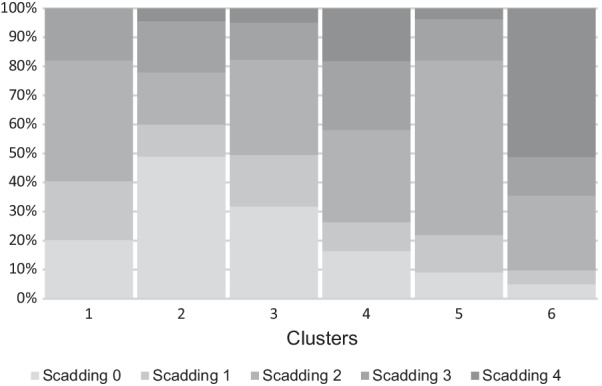
Distribution of Scadding stages 0–4 in each cluster. The representation of each Scadding stage in a cluster by percent is shown for all six clusters

**Fig. 3:**
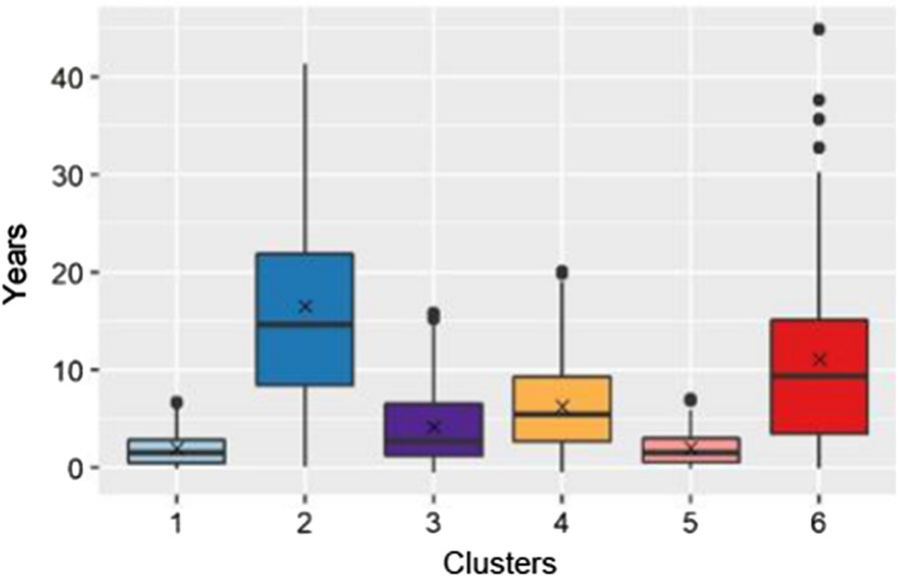
Comparison of duration of disease in years among clusters. For each cluster, median and IQR are shown by boxplots and means are shown by x in the center of boxplots. Potential outliers are indicated by distinct points

**Fig. 4:**
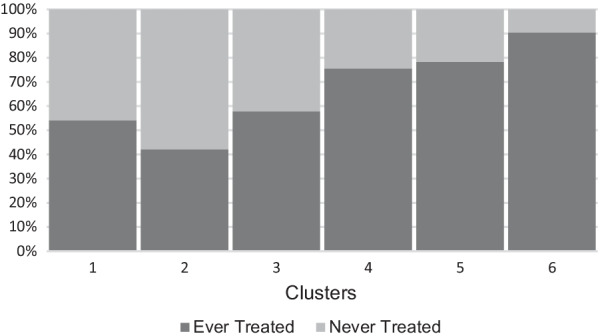
Distribution of cases treated with non-corticosteroid immunosuppression in each cluster. Percent of individuals who ever received immunosuppressive treatment are represented in dark gray, while percent of individual who have never received immunosuppressive treatment are in light gray

Six clusters were identified by model-based clustering. Based on the variable importance scores from the VarSelLCM model, the six variables most important for clustering in descending order were: FEV1pp, FVCpp, duration of disease, FEV1/FVC, Scadding stage and treatment status. The distributions of these variables are presented in Figs. 1, 2, 3, 4. We evaluated differences across clusters in these variables as noted in Table 2. We describe specific abnormalities by cluster in Fig. 6a, b.

**Table 2:** Differences in clinical characteristics across phenotypes

Characteristic^1,2^	1, N = 103	2, N = 45	3, N = 111	4, N = 114	5, N = 88	6, N = 93	q-value^3^
FEV1pp	104.4 (6.5) _E_	105.7 (11.5) _E_	91.8 (4.8) _D_	80.3 (6.1) _C_	71.2 (5.8) _B_	53.2 (12.4) _A_	**< 0.001**
FVCpp	104.3 (6.6) _E_	107.9 (12.2) _E_	87.5 (5.3) _C_	89.5 (8.7) _D_	72.2 (5.3) _B_	66.3 (15.2) _A_	**< 0.001**
FEV1/FVC	79.1 (4.8) _D_	76.4 (5.5) _C_	82.5 (4.0) _E_	70.3 (5.4) _B_	77.5 (6.2) _C,D_	63.5 (13.6) _A_	**< 0.001**
Duration of disease (years)	2.0 (1.7) _A_	16.5 (9.9) _E_	4.2 (3.8) _B_	6.3 (4.8) _C_	2.0 (1.8) _A_	11.1 (9.5) _D_	**< 0.001**
Ever treatment	39 (54.2%) _A_	16 (42.1%) _A_	52 (57.8%) _A_	74 (75.5%) _B_	54 (78.3%) _B,C_	75 (90.4%) _C_	**< 0.001**
Scadding stage							
0	19 (20.2%) _B,C_	22 (48.9%) _D_	32 (31.7%) _C,D_	18 (16.4%) _B_	7 (9.0%) _A,B_	4 (4.9%) _A_	**< 0.001**
1	19 (20.2%) _A_	5 (11.1%) _A_	18 (17.8%) _A_	11 (10.0%) _A_	10 (12.8%) _A_	4 (4.9%) _A_	
2	39 (41.5%) _B_	8 (17.8%) _A_	33 (32.7%) _A,B_	35 (31.8%) _A,B_	47 (60.3%) _C_	21 (25.6%) _A,B_	
3	17 (18.1%) _A_	8 (17.8%) _A_	13 (12.9%) _A_	26 (23.6%) _A_	11 (14.1%) _A_	11 (13.4%) _A_	
4	0 (0.0%) _A_	2 (4.4%) _A_	5 (5.0%) _A_	20 (18.2%) _B_	3 (3.8%) _A_	42 (51.2%) _C_	
BMI	28.8 (5.2) _A_	28.8 (6.0) _A_	31.8 (7.4) _B_	29.7 (6.1) _A_	32.9 (7.4) _B_	30.7 (7.3) _A,B_	**< 0.001**
Age at diagnosis	47.4 (11.0) _B_	41.0 (11.6) _A_	47.8 (10.5) _B_	47.2 (11.2) _B_	49.6 (9.7) _B_	43.7 (10.7) _A_	**< 0.001**
Female	56 (54.4%) _A,B_	30 (66.7%) _B_	70 (63.1%) _B_	47 (41.2%) _A_	47 (53.4%) _A,B_	39 (41.9%) _A_	**0.004**
Lofgren’s	6 (5.8%) _A_	3 (6.7%) _A_	6 (5.4%) _A_	0 (0.0%) _A_	1 (1.1%) _A_	1 (1.1%) _A_	**0.016**
Ever smoker	31 (30.4%) _A,B_	19 (42.2%) _A,B_	30 (27.0%) _A_	31 (27.4%) _A_	30 (34.1%) _A,B_	44 (47.3%) _B_	**0.024**
Race							
White	90 (90.0%) _A_	33 (75.0%) _A_	95 (86.4%) _A_	91 (79.8%) _A_	61 (72.6%) _A_	73 (81.1%) _A_	**0.025**
Black	7 (7.0%) _A_	11 (25.0%) _B_	13 (11.8%) _A,B_	23 (20.2%) _B_	19 (22.6%) _B_	17 (18.9%) _A,B_	
Asian	1 (1.0%)	0 (0.0%)	1 (0.9%)	0 (0.0%)	2 (2.4%)	0 (0.0%)	
American Indian	1 (1.0%)	0 (0.0%)	0 (0.0%)	0 (0.0%)	1 (1.2%)	0 (0.0%)	
Other	1 (1.0%)	0 (0.0%)	1 (0.9%)	0 (0.0%)	1 (1.2%)	0 (0.0%)	
Extrapulmonary Involvement	43 (41.7%)	21 (46.7%)	53 (47.7%)	62 (54.4%)	42 (47.7%)	38 (40.9%)	0.461
Cardiac	5 (4.9%)	6 (13.3%)	14 (12.6%)	23 (20.2%)	11 (12.5%)	12 (12.9%)	0.077
No. of organs	1.6 (1.0)	1.6 (1.1)	1.7 (1.1)	1.8 (1.0)	1.7 (1.0)	1.6 (1.0)	0.646

^1^Data presented: n (%); mean (SD)

^2^Statistical tests performed: Fisher’s exact test; Welch’s one-way ANOVA. For pairwise comparisons, 2-sample t-tests and logistic regression were performed; groups sharing the same letter (A, B, C, D, E) do not have significantly different means or proportions (q > 0.05)

^3^False discovery rate correction for multiple testing

For lung function (Fig. 1), mean FEV1pp and FVCpp were highest in cluster 1 (104.4 and 104.3 respectively) and cluster 2 (105.7 and 107.9). The highest mean FEV1/FVC ratio was present in cluster 3 (82.5). The clusters with lowest mean FEV1pp and FVCpp included cluster 5 (71.2 and 72.2) and cluster 6 (53.2 and 66.3). The clusters with significantly lower mean FEV1/FVC ratios included cluster 4 (70.3) and cluster 6 (63.5). Overall, cluster 6 had the lowest spirometry values out of all the clusters, although the distribution of the interquartile range (IQR) was broad: FEV1pp (46–62), FVCpp (55–76), FEV1/FVC (55–72).

While each cluster included representation of all five Scadding stages (Fig. 2), differences in the percentages were apparent. Cluster 1 was predominantly composed of Scadding stage 2 (41.5%), while cluster 2 was predominated stage 0 (48.9%). Cluster 5 was mostly Scadding stage 2 (60.3%), while cluster 6 had a majority Scadding stage 4 (51.2%). Clusters 3 and 4 contained no one prominent Scadding stage.

Differences in duration of disease were noted (Fig. 3) with clusters 1 (2) and cluster 5 (2) having significantly shorter mean durations of disease. Cluster 2 (16.5) and cluster 6 (11.1) had the longest mean durations of disease; however, the IQR were broad for these clusters: cluster 2 (8.4–21.9) and cluster 6 (3.4–15.1). Clusters 3 (4.2) and 4 (6.3) had intermediate mean durations. Significantly more individuals were on treatment in clusters 4, 5, 6 compared to clusters 1, 2, 3 (Fig. 4).

### Clinical characteristics differ between phenotypes

We evaluated the other variables entered in the cluster analysis to determine differences between clusters (Table 2, expanded table in Additional file 1: Table E1). Figure 6a, b represents specific abnormalities by cluster. In addition to the six variables mentioned above, BMI, age at diagnosis, gender, Lofgren’s syndrome, smoking status and race differed significantly. Specifically, clusters 3 and 5 had higher average BMI than clusters 1, 2, 4, while clusters 2 and 3 had more females compared to more males in clusters 4 and 6. Cluster 6 contained more smokers compared to 3 and 4. More Black individuals were in clusters 2, 4, 5 versus cluster 1. Finally, individuals in clusters 2 and 6 were younger at diagnosis versus those in clusters 1, 3, 4 and 5. Interestingly, specific extrapulmonary organ involvement did not differ across clusters, however there was a trend toward significance for cardiac involvement. When the cluster analysis was rerun using “yes/no” for extrapulmonary involvement, there were still no differences across clusters; additionally, analysis yielded the same results with the same six clusters.

### Wasfi score association with phenotypes

**Fig. 5:**
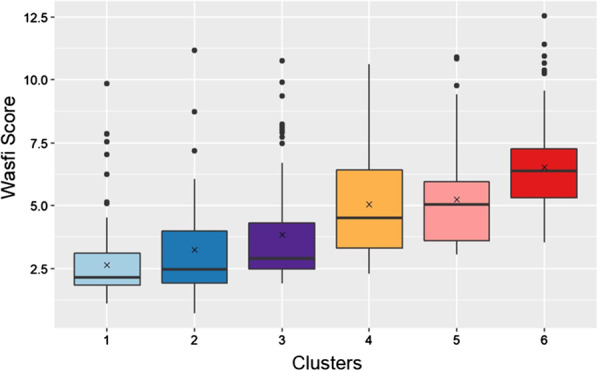
Wasfi Scores by Cluster. For each cluster, median and IQR are shown by boxplots and means are shown by x in the center of boxplots. Higher scores indicate greater severity

We evaluated the association of the Wasfi severity score with each of our clusters. The mean Wasfi score differed significantly across the six clusters (q < 0.001, Fig. 5), with clusters 4, 5, 6 (mean scores of 5, 5.2 and 6.5 respectively) significantly higher than cluster 1, 2, 3 (mean scores 2.6, 3.2 and 3.8).

### Phenotypes of sarcoidosis disease severity

**Fig. 6:**
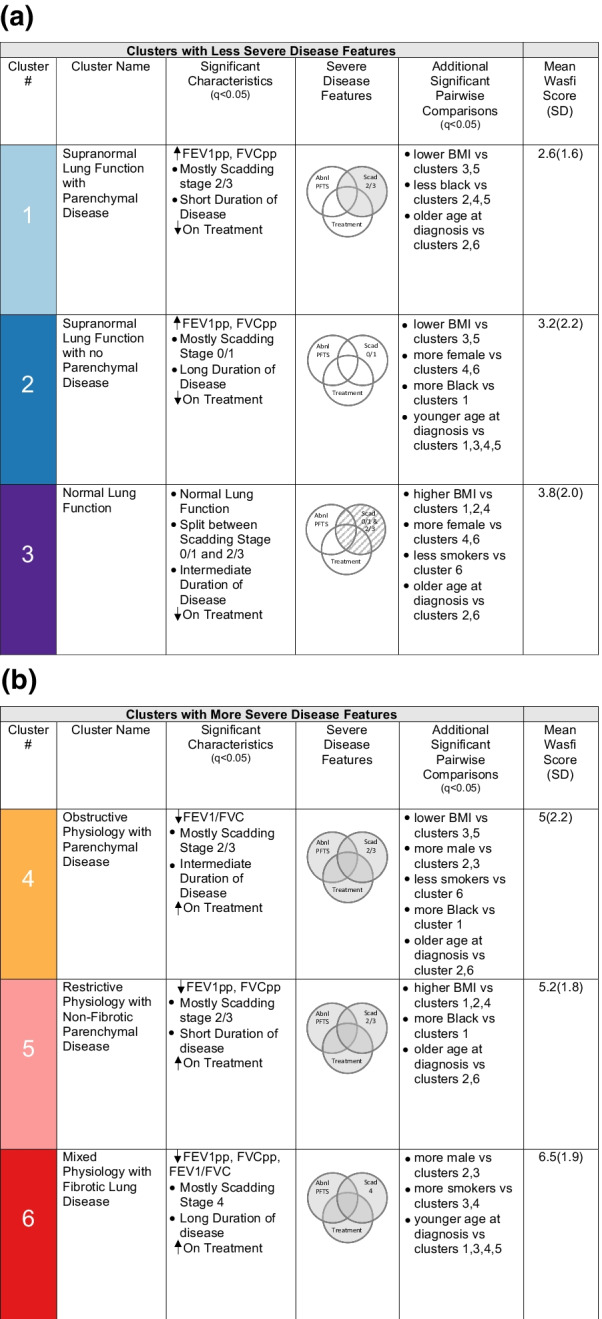
**a** Cluster Descriptions by Less Severe Disease Features. **b** Cluster Descriptions by More Severe Disease Features. The first column describes the cluster number, and the second column describes the cluster name. The third column includes significant differences in the six most important variables for clustering; arrows indicate a significant difference between less severe clusters (1, 2, 3) and more severe clusters (4, 5, 6) (q < 0.05). The fourth column shows which severe disease features are present in clusters; shading in the Venn diagram indicates that the majority of individuals had that particular disease feature; partial shading indicates half of individuals had the disease feature. The fifth column describes significant pairwise differences between clusters (q < 0.05). Finally, the sixth column describes the mean Wasfi score for that cluster

Based on our cluster analyses, and their associations with clinical variables and Wasfi score analyses, we categorized the clusters based on disease severity and other disease findings. Overall, it appears that the clusters reflect less severe (clusters 1, 2, 3) and severe pulmonary disease manifestations (clusters 4, 5, 6) (Fig. 6a, b). Specifically, individuals in clusters 4, 5, 6 had at least one lung function parameter lower than normal and required more treatment versus those in clusters 1, 2, 3. The individuals in clusters 4, 5, 6 also had unique patterns of lung function abnormalities, specifically obstructive, restrictive and mixed patterns, respectively. Scadding stage was less distinctly distributed between the severe and less severe clusters, although the severe phenotypes had less stage 0/1 disease and cluster 6 had more stage 4 disease, consistent with a fibrotic phenotype. Severe clusters 4 and 6 had more males than less severe clusters 2 and 3. The rest of the variables did not demonstrate a clear distinction between severe and less severe clusters. Based on lung function and radiological differences, we named the clusters as noted in Fig. 6a, b.

## Discussion

There is a pressing need for sarcoidosis phenotypes that can identify those with or at risk for severe disease and to classify them for research studies. We used cluster analysis on clinical characteristics to define sarcoidosis phenotypes and found that common clinical variables contributed most to the clustering, including spirometry, disease duration, Scadding stage and immunosuppressive treatment. Unexpectedly, we defined six distinct pulmonary phenotypes that included severe and less severe disease manifestations but did not differ in extrapulmonary organ involvement. The three less severe phenotypes were classified as supranormal lung function with parenchymal disease, supranormal lung function with no parenchymal disease and normal lung function. The three severe phenotypes included obstructive physiology with parenchymal disease, restrictive physiology with non-fibrotic parenchymal disease and mixed physiology with fibrotic lung disease. Interestingly, male gender was predominant in two of the more severe clusters while females predominated the less severe clusters. Unsurprisingly, Black individuals made up a greater proportion in two of the severe clusters, and a similar proportion in one of the less severe clusters. Finally, we compared our cluster phenotypes with a previously determined assessment of disease severity developed by our group, the Wasfi score, and found that our less severe clusters had lower scores, while the more severe clusters had higher scores.

Our clusters describe pulmonary disease phenotypes despite the inclusion of other organ involvement. Our unique phenotypes suggest subgroups of pulmonary sarcoidosis based on different lung function and radiographic abnormalities. Our severe phenotypes clusters 4, 5, 6 had lower lung function that was obstructive, restrictive and mixed respectively and were associated with different Scadding stages. Various lung function abnormalities have been implicated with worse outcomes in sarcoidosis, specifically FVC < 80%, FEV1 < 50% and a vital capacity less than 1.5 L. [5, 24–26] Previous studies have shown limited correlation between initial Scadding stage and subsequent clinical recovery or lung function [4, 26–28] except for Scadding Stage 0 and 4, which have been associated with good and poor prognosis respectively. Indeed, we identified more Scadding stage 4 in our cluster with the worst lung function and Scadding stage 0/1 in our cluster with supranormal lung function. However, Scadding stages 2/3 were represented in both severe and less severe clusters, which supports that Scadding stage is a poor disease predictor except at the extremes. This is not surprising as other studies have found that extremes in Scadding stage, and not stages 2/3, tend to be more predictive of disease course/severity; this is likely due to the vast spectrum of disease abnormalities represented by stage 2/3. The need for treatment is often associated with chronic respiratory impairment [24, 29]. Those who are initially treated are more likely to require treatment at follow-up and relapse with treatment cessation [7, 27]. We find a clear association with treatment and severe and less severe clusters with more individuals in the severe groups on non-corticosteroid immunosuppressive treatment. Individuals who were diagnosed at earlier ages had the longest durations of disease, which did not correlate with disease severity. Clusters that share a similar duration of disease allow identification of distinct phenotypes at a similar point in time without having longitudinal data. For instance, clusters 1 and 5 share a similar short disease duration (average 2 years), but it is obvious these are two distinct phenotypes with cluster 1 exhibiting less severe disease than cluster 5. To determine how the clusters change over time would require longitudinal data, which we did not include; it is possible that individuals may move from one phenotype to another at different time points.

While some of our findings support prior studies, others were unexpected. For example, males were the majority of our severe clusters 4 and 6, while females were the majority in the less severe clusters 2 and 3. These finding are somewhat at odds with prior studies where women, especially Black women, have higher mortality [30, 31] and more severe organ involvement [32, 33]. However, these findings may support studies suggesting that males have a more chronic course than females [25]. Unexpectedly, our less severe cluster 2 had a greater frequency of Black individuals than the other less severe clusters although severe clusters 4 and 5 also had more Black individuals. There is significant literature supporting that Black individuals have more severe disease requiring treatment and higher associated mortality [4, 7, 30, 31, 34]. Most of our participants in this study were White, which may have impacted the results, although they may also suggest that severe sarcoidosis affects all races. It is well documented that there is a decreased prevalence of sarcoidosis among smokers [35–38]. However, we found that our most severe cluster 6 had the highest percent smokers, suggesting that disease severity may be worse for smokers. This is seen in other pulmonary granulomatous diseases such as chronic beryllium disease and hypersensitivity pneumonitis, where smokers have worse pulmonary function and require more treatment compared to never smokers despite having a lower prevalence of disease [39, 40]. Cluster 6 individuals were also younger at diagnosis and had longer disease duration, which is consistent with the fact that fibrosis is associated with a prolonged duration of disease [31]. Interestingly, this is not the case with cluster 2, which also has a younger age of diagnosis and longer duration of disease. The may reflect that these two clusters represent two different phenotypes. Additionally, cluster 6 had the highest percentage of males; this is an interesting observation as males are often diagnosed at a younger age, and may have more chronic disease, more stage IV fibrotic disease, and higher mortality from fibrosis. [32, 41, 42].

We compared our phenotypes to another phenotyping method developed by our group; the Wasfi severity score gives a numerical severity index developed to codify expert opinion [8]. Our three severe disease clusters were associated with higher Wasfi severity scores. This was not surprising as the features clustered in our severe phenotypes, abnormal spirometry and treatment, are part of the Wasfi score. Unlike the Wasfi score, non-pulmonary organ involvement, including cardiac and neurological, did not contribute to our clusters/phenotypes. Our treatment variable timeframe was different than that used in the Wasfi severity score because we wanted to approximate ever treatment in our cohort using a 5-year timeframe instead of the 30-day timeframe used in Wasfi; however, we found that the Wasfi 30-day treatment variable was highly correlated with our 5-year treatment variable. Other studies have used cluster analyses to define phenotypes in sarcoidosis [13–15, 43]. However, in contrast to our study, they used organ specific variables to produce organ-based phenotypes. Unexpectedly in our study, extrapulmonary organ variables did not contribute to the clustering of our phenotypes; while cardiac involvement trended toward being significantly different between clusters, we did not see more cardiac involvement in our severe phenotypes as we anticipated. This might be due to low extrapulmonary organ frequencies in our cohort, although performing cluster analysis using only the presence/absence of extrapulmonary disease did not affect our results. Additionally, Schupp et al. included similarly low extrapulmonary organ frequencies in a large European cohort to develop organ-based phenotypes [13]. Our results may suggest that extrapulmonary organ involvement is not a predominant phenotype when clinically relevant pulmonary variables are included; pulmonary involvement is overwhelmingly the most commonly involved organ in sarcoidosis and results in significant morbidity and mortality [44]. A study by Rodrigues et al., used factor analysis with clinical input variables similar to those used in our study to analyze a Brazilian cohort and found four phenotypes [45]. Similar to our results, they found a phenotype characterized by fibrosis/Scadding stage 4 and decreased lung function parameters as well as one marked by airflow obstruction. Despite the differences in our statistical methods, the similarities in our resultant phenotypes do suggest consistency of results as well as demonstration of the importance of including clinically relevant variables.

While we are a major sarcoidosis referral center, we often see more complicated cases, including severe pulmonary and extrapulmonary disease; this may have biased our cohort towards more severe disease. While this could have impacted extrapulmonary disease severity, our rates of other organ involvement were similar to other studies [13]. Our study emphasizes the need for inclusion of clinically relevant measures of extrapulmonary disease severity, like arrhythmias or ejection fraction for cardiac disease, if the goal is to evaluate other organ specific or overall severe disease. Additionally, other clinical markers of disease severity such as lymphopenia were not available in our cohort. We did not have patient reported outcomes or symptoms, which could provide information missed by objective measurements in a phenotyping paradigm designed to assess disease course and therapeutic intervention; organ specific phenotypes may or may not be helpful for these applications. We did not have longitudinal data to test the stability of our clusters over time, although we were able to infer some longitudinal information based on duration of disease as described above; additionally, previous studies have noted stability in FVC, FEV1 and Scadding stage over a 2-year period suggesting that many of our clusters may remain the same over this time frame [28]. We intentionally chose not to include corticosteroid therapy as a variable because we find that many individuals with sarcoidosis are over-treated with corticosteroids; however, we cannot completely rule out that including corticosteroid may have changed our clustering results. For future directions, a larger cohort followed longitudinally would allow for a deeper analysis of treatment types, treatment failure, the effects of age and sex, and extrapulmonary organ severity based on objective measures. This will allow us to validate the findings we have found in this manuscript. Finally, given the inherent uncertainty in statistical techniques we cannot say that there are definitively only six sarcoidosis clusters, which is an issue that applies to all forms of cluster analyses.

## Conclusion

In conclusion, this study is novel in that it uses the objective method of cluster analyses to clinically phenotype sarcoidosis patients with easily obtained clinical characteristics beyond organ involvement. It demonstrates the importance of clinical variables to define clinically relevant phenotypes and suggests that inclusion of longitudinal data may add to the model, which is a plan for future directions. Furthermore, these pulmonary phenotypes were further categorized into less severe and severe phenotypes. Specifically, these phenotypes may help clinicians identify individuals who are more likely to have severe disease in phenotypes 4, 5, and 6, while being able to offer reassurance to those in phenotypes 1–3. For phenotypes 1 and 3 with shorter time since diagnosis, there could be important differences among the less severe phenotypes, which could be elucidated in longitudinal follow-up in future studies. The methods of this study suggest an approach for other organ specific phenotyping. Finally, these phenotypes have the potential to help identify subgroups in this heterogeneous disease that may have implications in follow-up, prognosis and possibly interventions.

## Supplementary Information

**Additional file 1:** Differences in Individual Organ Involvement across Phenotypes.

## Data Availability

The datasets used and/or analyzed during the current study are available from the corresponding author on reasonable request.

## Abbreviations

BMI: Body mass index
FDR: False-discovery-rate
FET: Fisher’s exact test
FEV1pp: Forced expiratory volume in one second percent predicted
FVCpp: Forced vital capacity percent predicted
FEV1/FVC: Forced expiratory volume in one second/forced vital capacity
ICL: Integrated completed likelihood
IQR: Interquartile range
NJH: National Jewish Health
