# Supplementary material for: Clinical phenotyping in sarcoidosis using cluster analysis
Source: Respir Res. 2022 Apr 9;23:88. doi: 10.1186/s12931-022-01993-z (PMC8994095; doi:10.1186/s12931-022-01993-z)
Supplement: Supplementary file 1 — Additional file 1: Differences in Individual Organ Involvement across Phenotypes. [file 12931_2022_1993_MOESM1_ESM.docx]

**Supplemental Material**

Table E1. Differences in Individual Organ Involvement across Phenotypes

| Characteristic^1,2^ | 1,  N = 103 | 2,  N = 45 | 3,  N = 111 | 4,  N = 114 | 5,  N = 88 | 6,  N = 93 | q-value^3^ |
| --- | --- | --- | --- | --- | --- | --- | --- |
| **Liver** | 12 (11.7%) | 1 (2.2%) | 6 (5.4%) | 10 (8.8%) | 8 (9.1%) | 3 (3.2%) | 0.299 |
| **Bones and Joints** | 3 (2.9%) | 0 (0.0%) | 5 (4.5%) | 8 (7.0%) | 2 (2.3%) | 1 (1.1%) | 0.345 |
| **Kidneys** | 0 (0.0%) | 0 (0.0%) | 5 (4.5%) | 2 (1.8%) | 1 (1.1%) | 1 (1.1%) | 0.345 |
| **Extra thoracic Lymph Node** | 4 (3.9%) | 6 (13.3%) | 8 (7.2%) | 6 (5.3%) | 8 (9.1%) | 3 (3.2%) | 0.345 |
| **Eye** | 12 (11.7%) | 8 (17.8%) | 15 (13.5%) | 10 (8.8%) | 8 (9.1%) | 5 (5.4%) | 0.362 |
| **Bone Marrow** | 0 (0.0%) | 1 (2.2%) | 1 (0.9%) | 0 (0.0%) | 1 (1.1%) | 2 (2.2%) | 0.440 |
| **Lungs** | 100 (97.1%) | 41 (91.1%) | 106 (95.5%) | 112 (98.2%) | 85 (96.6%) | 90 (96.8%) | 0.634 |
| **Skin** | 9 (8.7%) | 9 (20.0%) | 13 (11.7%) | 16 (14.0%) | 10 (11.4%) | 11 (11.8%) | 0.752 |
| **Calcium/Vit D Dysregulation** | 14 (13.6%) | 2 (4.4%) | 11 (9.9%) | 11 (9.6%) | 7 (8.0%) | 9 (9.7%) | 0.810 |
| **Spleen** | 3 (2.9%) | 1 (2.2%) | 4 (3.6%) | 3 (2.6%) | 6 (6.8%) | 2 (2.2%) | 0.810 |
| **Ear Nose Throat** | 4 (3.9%) | 1 (2.2%) | 1 (0.9%) | 3 (2.6%) | 2 (2.3%) | 1 (1.1%) | 0.810 |
| **Muscle** | 0 (0.0%) | 0 (0.0%) | 2 (1.8%) | 2 (1.8%) | 1 (1.1%) | 2 (2.2%) | 0.840 |
| **Neuro** | 3 (2.9%) | 1 (2.2%) | 1 (0.9%) | 4 (3.5%) | 2 (2.3%) | 3 (3.2%) | 0.870 |
| **Parotid/Salivary Gland** | 0 (0.0%) | 0 (0.0%) | 2 (1.8%) | 1 (0.9%) | 1 (1.1%) | 1 (1.1%) | 0.873 |
| **Other Organs** | 3 (2.9%) | 3 (6.7%) | 4 (3.6%) | 2 (1.8%) | 2 (2.3%) | 3 (3.2%) | 0.810 |
|  |  |  |  |  |  |  |  |
| ^1^Data presented: n (%)  ^2^Statistical tests performed: Fisher's exact test; Welch's one-way ANOVA.  ^3^False discovery rate correction for multiple testing | | | | | | | |
